# Application of an Exploratory Knowledge-Discovery Pipeline Based on Machine Learning to Multi-Scale OMICS Data to Characterise Myocardial Injury in a Cohort of Patients with Septic Shock: An Observational Study

**DOI:** 10.3390/jcm10194354

**Published:** 2021-09-24

**Authors:** Bernardo Bollen Pinto, Vicent Ribas Ripoll, Paula Subías-Beltrán, Antoine Herpain, Cristina Barlassina, Eliandre Oliveira, Roberta Pastorelli, Daniele Braga, Matteo Barcella, Laia Subirats, Julia Bauzá-Martinez, Antonia Odena, Manuela Ferrario, Giuseppe Baselli, Federico Aletti, Karim Bendjelid, on behalf of the Shockomics Consortium

**Affiliations:** 1 Department of Acute Medicine, Geneva University Hospitals, 1205 Geneva, Switzerland; karim.bendjelid@hcuge.ch; 2 Unit of Digital Health, Eurecat, Centre Tecnològic de Catalunya, 08290 Barcelona, Spain; vicent.ribas@eurecat.org (V.R.R.); paula.subias@eurecat.org (P.S.-B.); laia.subirats@eurecat.org (L.S.); 3 Department of Intensive Care, Erasme University Hospital—Université Libre de Bruxelles, 1070 Brussels, Belgium; antoine.herpain@erasme.ulb.ac.be; 4 Fondazione Filarete, 20139 Milan, Italy; mariacristinabarlassina@gmail.com (C.B.); daniele.braga@humanitasresearch.it (D.B.); barcella.matteo@hsr.it (M.B.); 5 Dipartimento di Scienze della Salute, Università degli Studi di Milano, 20146 Milan, Italy; 6 Proteomics Platform, Parc Científic de Barcelona, 08028 Barcelona, Spain; eoliveira@pcb.ub.es (E.O.); juliabauza1994@gmail.com (J.B.-M.); maodena@pcb.ub.es (A.O.); 7 IRCCS—Istituto di Ricerche Farmacologiche Mario Negri, 20156 Milan, Italy; roberta.pastorelli@marionegri.it; 8 Department of Electronics, Information and Bioengineering, Politecnico di Milano, 20133 Milano, Italy; manuela.ferrario@polimi.it (M.F.); giuseppe.baselli@polimi.it (G.B.); 9 Instituto de Ciência e Tecnologia, Universidade Federal de São Paulo (UNIFESP), São Paulo 12231-280, Brazil; aletti@unifesp.br

**Keywords:** machine learning, feature selection, septic cardiomyopathy, myocardial injury, septic shock

## Abstract

Currently, there is no therapy targeting septic cardiomyopathy (SC), a key contributor to organ dysfunction in sepsis. In this study, we used a machine learning (ML) pipeline to explore transcriptomic, proteomic, and metabolomic data from patients with septic shock, and prospectively collected measurements of high-sensitive cardiac troponin and echocardiography. The purposes of the study were to suggest an exploratory methodology to identify and characterise the multiOMICs profile of (i) myocardial injury in patients with septic shock, and of (ii) cardiac dysfunction in patients with myocardial injury. The study included 27 adult patients admitted for septic shock. Peripheral blood samples for OMICS analysis and measurements of high-sensitive cardiac troponin T (hscTnT) were collected at two time points during the ICU stay. A ML-based study was designed and implemented to untangle the relations among the OMICS domains and the aforesaid biomarkers. The resulting ML pipeline consisted of two main experimental phases: recursive feature selection (FS) assessing the stability of biomarkers, and classification to characterise the multiOMICS profile of the target biomarkers. The application of a ML pipeline to circulate OMICS data in patients with septic shock has the potential to predict the risk of myocardial injury and the risk of cardiac dysfunction.

## 1. Introduction

Sepsis is a life-threatening organ dysfunction caused by a dysregulated immune response to infection [1] and septic shock is its most severe form associated with higher mortality [2]. More than half of the patients with septic shock present elevated levels of circulating cardiac biomarkers, such as troponin (herein referred as myocardial injury) and some degree of impairment in echocardiographic indices of diastolic and/or systolic function (herein referred as cardiac dysfunction), conditions commonly grouped under the terminology of septic cardiomyopathy (SC) [3]. Patients with SC have higher mortality rates than those without it [3].

At present, there is no therapy specially targeting SC. While positive inotropes, mainly dobutamine, are used clinically to ameliorate cardiac functions and improve both cardiac output and systemic oxygen delivery, excessive β-adrenergic stimulation can be associated with harm [4]. Initial enthusiasm with Levosimendan, a calcium sensitizer [5], has not been confirmed in a subsequent large randomised-control trial [6], although the latter did not target to treat overt SC, per se. One likely reason for the lack of successful interventions targeting SC is that we fail to understand the root causes of heart affection (myocardial injury and cardiac dysfunction) in patients with septic shock. The underlying pathophysiology is certainly complex and studies performed in sub-optimal animal models have proposed a number of events and pathways [7] that have rarely been confirmed in human subjects.

The characterisation of patients at the molecular level is a promising approach to identify pathophysiological mechanisms and specific targets for new therapeutic interventions in critically-ill patients with a particular condition [8]. For example, we have previously shown that changes in the metabolome (lipidome in particular) and transcriptome may play a relevant role in early recovery of organ dysfunction in patients with septic shock [9,10].

In this work, we used a machine learning (ML) pipeline to investigate the prospects of its application to analyse transcriptomic, proteomic and metabolomic data gathered at two time points during Intensive Care Unit (ICU) stay in patients with septic shock and prospectively collected measurements of high-sensitive cardiac troponin and echocardiography. Our primary aim was to identify and characterise the multiOMICs profile of myocardial injury in patients with septic shock. Second, we aimed at identifying if a distinct profile exists when cardiac dysfunction in patients is associated with myocardial injury.

## 2. Materials and Methods

### 2.1. Study Design and Participants

This manuscript follows the STROBE guidelines for reporting observational studies (Table S1) [11].

This study is part of the multicentre prospective observational trial “ShockOmics” (ClinicalTrials.gov Identifier NCT02141607) [12]. Patients were recruited in the Intensive Care Units (ICU) of Hopitaux Universitaires de Genève (Geneva, Switzerland) and Hôpital Erasme—Cliniques Universitaires de Bruxelles (Brussels, Belgium). The study was approved by the Geneva Regional Research Ethics Committee (study number 14-041) and the Ethical committee of Hôpital Erasme-Université Libre De Bruxelles (study number P2014/171). Informed consent was obtained from the patients or their representatives.

As detailed elsewhere [12], we included consecutive adult (>18 years old) patients, admitted for septic shock in the ICUs of two University Hospitals, with an admission SOFA score ≥6, and an arterial lactate ≥2 mmol/L. Although septic shock was defined according to the recommendations and international guidelines at the time of inclusion [13], all patients fulfil the criteria of Sepsis-3 [1]. Peripheral blood samples for OMICS analysis and measurements of high-sensitive cardiac troponin T (hscTnT) were collected within 16 h of ICU admission (T1) and 48 h after admission (T2). A certified intensivist performed an echocardiography at the same time points. Left ventricle ejection fraction (LVEF) was measured in apical view, according to the biplane modified Simpson’s method.

We excluded patients expected to die within 24 h after ICU admission and with terminal illness; those receiving more than four units of red blood cells or >1 fresh frozen plasma transfused; with active haematological malignancy, metastatic cancer, chronic immunodepression, pre-existing end-stage renal disease requiring renal replacement therapy, recent cardiac surgery, and Child-Pugh C cirrhosis. The main reason for our exclusion criteria was to avoid confounding factors that would make it difficult to distinguish at transcriptomic, proteomic, and metabolomic levels what is due to septic shock and what to the comorbidities. We also excluded patients who did not have data in the three OMICS domains. Furthermore, we prospectively planned at least two time points in our study using expensive technologies, hence the two exclusion criteria of “terminal illness” and high risk of death within 24 h of ICU admission. Demographic and clinical characteristics in patients with and without myocardial injury (Table 1 and Table 2) were compared using Fisher’s exact test or Mann–Whitney U test, as appropriate.

### 2.2. OMICS Data

Blood samples were collected in EDTA tubes and treated as follows:

- For transcriptomics: after adding 400 µL of 2X Denaturing solution (Ambion, Austin, Texas, USA) to an equal volume of blood, samples were stored at −20 °C until analysis;
- For proteomics, metabolomics and hscTnT quantification: after adding 900 µL of a protease inhibitor solution (Roche Applied Science, Penzberg, Germany) to 6 mL of blood, 0.5 mL plasma aliquots were obtained by two-step centrifugation and stored at −80 °C until analysis.

All subsequent analytic steps were performed in batches.

### 2.3. Transcriptomics Analysis

As detailed elsewhere [14], total RNA was extracted from blood samples with a MirVana Paris Kit (Applied Biosystems, Waltham, Massachusetts, United States)and treated with Turbo DNA-free Kit (Ambion, Austin, Texas, USA). RNA Quality was assessed on Agilent Bioanalyzer with the RNA 6000 Nano Kit (Agilent, Santa Clara, CA, USA) and samples were considered suitable for processing if RNA Integrity Number was greater than 7.5. Sequencing libraries were prepared with the TruSeq Stranded Total RNA with Ribo-Zero Globin Kit (Illumina, San Diego, CA, USA) using 800 ng of total RNA. Final libraries were validated with the Agilent DNA1000 kit (Agilent, Santa Clara, California, United States) and sequenced on a HiSeq2500 platform producing 50 × 2 bp paired end reads. High quality paired-end reads were aligned to the human reference genome (GRCh38) using STAR (v2.5.2b) (Github, San Francisco, CA, USA) [15] emitting only uniquely mapping reads. Reads were assigned to genes with featureCounts (v1.5.1) [16] using the GENCODE (v.25) (GENCODE reference annotation for the human and mouse genomes”) primary assembly gene transfer file (GTF) as the reference annotation file for genomic features boundaries.

DESeq2 package (Bioconductor) [17] built-in functions were used to perform data pre-processing and export of normalised counts.

### 2.4. Proteomics Analysis

The proteomics analysis was performed using Tandem Mass Tag, TMT-10plex (Thermo Scientific) technique. Firstly, immunoaffinity depletion of highly abundant proteins from plasma samples was performed using IgY14 Seppro^®^ column (Sigma—St. Louis, MO, USA). Eluted proteins were reduced, alkylated, and double trypsin digested. Seven TMT-10plex experiments were performed. After peptide labelling, samples were subjected to high pH fractionation with a high pH reversed phase peptide fractionation kit (Pierce, ref. 84868—Thermo Fisher, Waltham, MA, USA). A total of eight fractions from each TMT-10plex batches were analysed using an Orbitrap Fusion Lumos™ Tribrid mass spectrometer (Thermo Scientific, Waltham, MA, USA). The mass spectrometer was operated in a data-dependent acquisition (DDA) mode. MS2-MS3 analysis was conducted with a top speed approach. Thermo Proteome Discover (v.2.1) Thermo Scientific, Waltham, MA, USA) was used to search with Sequest HT search engine against the Swiss-Prot human public database. For each TMT batch, eight raw files corresponding to the eight fractions injections from the MS analyses were used to perform a single search against this database. The quantification of proteins was conducted by summing, within each TMT™ 10plex experiment, the reporter ion intensities of unique peptides. Libra channel normalization was performed for each TMT™ 10plex experiment.

### 2.5. Metabolomics Analysis

We performed a targeted quantitative approach using a combined direct flow injection and liquid chromatography (LC) tandem mass spectrometry (MS/MS) assay (AbsoluteIDQ 180 kit, Biocrates, Innsbruck, Austria), as detailed elsewhere [9]. This method combines derivatisation and extraction of analytes with the selective mass-spectrometric detection using multiple reaction monitoring (MRM) pairs. Isotope-labelled internal standards are integrated into the platform for absolute quantification of metabolites. MRM detection was used for quantification applying spectra parsing algorithm integrated into the Metiq software (Biocrates Life Science AG, Innsbruck, Austria). Concentrations were calculated and evaluated by comparing measured analytes in a defined extracted ion count section to those of specific labelled internal standards or non-labelled ones, provided by the kit. This strategy allows simultaneous quantification of up to 186 metabolites. Metabolites were excluded from further analysis if: (1) fewer than 20% of missing values (non-detectable peak) for each quantified metabolite, (2) 50% of all sample concentrations for the metabolite had to be above the limit of detection (LOD). In total, 130 of the 186 metabolites were used for statistical analysis.

### 2.6. Definition of Myocardial Injury and Cardiac Dysfunction

Myocardial injury was defined as a circulating hscTnT level >14 ng/L, the 99th percentile upper reference limit of the assay, according to the Fourth universal definition of myocardial infarction [18]. Cardiac dysfunction was prospectively defined as a (LVEF) < 50% or treatment with positive inotropic drugs to improve cardiac output and tissue perfusion as judged necessary by the treating physician. Echocardiography image acquisition was performed by skilled Intensivists with a National Diploma of echocardiography. Analysis of the echocardiography images was performed by two assessors with a National Diploma of echocardiography and extensive teaching experience in echocardiography (KB and AH). Both assessors were blinded for the cardiac troponin measurements and OMICS results.

### 2.7. Multiscale Modelling of OMICS Data

Metabolomics and transcriptomics data have been previously published. Proteomics data has not. No analyses regarding the phenotypes of myocardial injury and cardiac dysfunction or the construction of multiOMICS models have been previously published for this cohort.

The ML pipeline presented in this paper is divided into two main experimental phases: FS and classification. The FS experiments started with the execution of tests to compare distributions where the data from our analysis pipeline has been further divided into six groups. Each group corresponds to its own dataset: transcriptomics at T1, transcriptomics at T2, proteomics at T1, proteomics at T2, metabolomics at T1, and metabolomics at T2. The FS phase started with a one-way ANOVA and a Kruskal–Wallis test to select the variables that yield a significant *p*-value and a reduced *q*-value [19], as appropriate. The normality of all OMICS data were assessed through the Shapiro–Wilk test and the variance homogeneity through Bartlett’s test. After comparing the distributions, the FS phase was completed with a recursive feature selection implemented with random forests. In this analysis, we also calculated the stability score for each biomarker, presented as a frequency (i.e., number of times a particular biomarker has been selected in the reported number of experiments). Due to the high dimensionality of the data, we propose to reduce first its dimensionality and later study the relation among the biomarkers. However, in case of working with lower dimensional data, the step of reducing dimensionality could be omitted.

The set of markers obtained in our FS phase was further analysed in an enrichment and pathway analysis. Particularly, the transcripts were analysed with Enrichr [20], proteins were analysed with Impala [21] and metabolites with MetaboAnalyst [22].

Since this is a knowledge discovery study, our main assumption here is that the biomarker sets yielding the best performances to assess myocardial injury and cardiac dysfunction (based on troponin and ejection fraction or an inotrope requirement) will also shed light on the pathophysiological processes involved in SC. For this reason, the set of biomarkers that result from the FS phase was used to create a set of classifiers to predict cardiac injury and dysfunction. In our case, we used logistic regression, classification trees (CART), and a support vector classifier (SVC) [23]. All of these methods are well suited for knowledge discovery from small-sized data sets for their interpretability and their execution time, which allow efficient implementations for training through leave-one-out cross-validation (LOOCV). The performance of each classifier has been evaluated through its accuracy, sensitivity, and specificity. The accuracy is strengthened by the *p*-value obtained in the McNemar’s test, and the sensitivity and specificity are shown together with their binomial confidence interval.

All of the analyses were performed using R and the main packages employed were: *stats*, *caret*, *randomForest*, *rpart*, and *e1071*.

## 3. Results

### 3.1. Characteristics of the Patients

**Figure 1:**
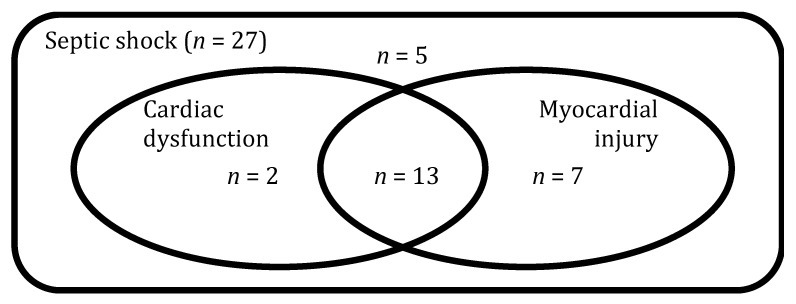
Septic cardiomyopathy phenotypes. Venn diagram depicting the number of patients with myocardial injury (hscTnT > 14 ng/L) and cardiac dysfunction (LVEF < 50% or cardiac dysfunction requiring treatment with inotropes). For more details, please refer to Materials and Methods. *n*—number of patients.

Between October 2014 and December 2015, we screened 529 patients for the whole ShockOmics study, of which 27 patients with septic shock were included in the present analysis (Figure S1). Twenty patients (74%) had an elevated troponin fitting the pre-defined criteria of myocardial injury (Figure 1). Of these, 13 (65%) patients presented with cardiac dysfunction. Eight patients were treated with positive inotropes (all with dobutamine). Table 1 and Table 2 resume the characteristics of the patients at ICU admission. Overall, ten patients (37%) had a respiratory infection and nine (33%) an abdominal infection.

Five (25%) patients with myocardial injury died in the ICU, while no patients died in the no-injury group (*p* = 0.143). At 100 days after admission, there were 7 (35%) deaths in the injury group and none (0%) death in the no-injury group (*p* = 0.168).

### 3.2. Multiscale Analysis

There were a total of 58,572 attributes, all numerical. Following the analysis pipeline outlined in the Materials and Methods presented above, we selected the biomarkers with a *p*-value < 0.05 for proteomics and metabolomics separately. Regarding transcriptomics, we also selected a *q*−value < 0.5 to further decrease the dimensionality of this set, which originally contained 8215 attributes. The normality of all OMICS data were assessed through the Shapiro–Wilk test with a Bonferroni correction of α = 0.05 and the variance homogeneity through Bartlett’s test. This study concluded that 58,514 attributes met the requirements to run one-way ANOVA whilst 58 did not. None of the 58 attributes analysed through the Kruskal–Wallis test presented a significant association with the aforesaid target biomarkers. As previously stated, we ran these analyses twice: the former focusing on the patients with myocardial injury in the whole cohort, and the latter focusing on the patients with cardiac dysfunction in patients presenting myocardial injury.

**Figure 2:**
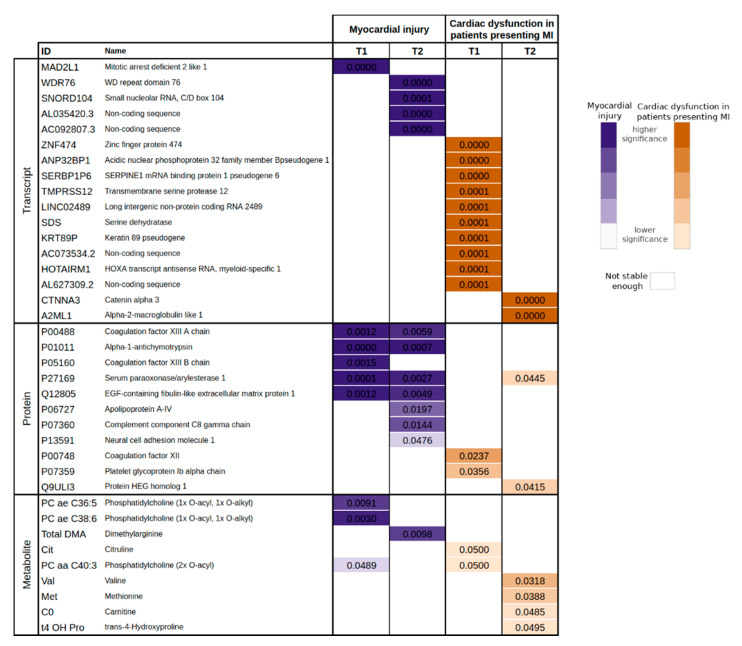
One-way ANOVA for the biomarkers related to myocardial injury in the whole cohort (MI: n_T1_ = 20 and n_T2_ = 19—not MI: n_T1_ = 7 and n_T2_ = 8), and to cardiac dysfunction in patients presenting myocardial injury (MI and CD: n_T1_ = 13 and n_T2_ = 9—MI and not CD: n_T1_ = 7 and n_T2_ = 10). The significance of each biomarker is interpreted by means of its p-value, where dark colours stand for higher significance (*p*-value < 1 × 10^−3^) and light colours for lower significance (max *p*-value 0.05). Blank cells represent biomarkers considered not stable enough in the FS for the specific time point and symptomatology.

Table 3 presents the list of markers related to myocardial injury resulting from this analysis. Next, we applied the same ML methodology to identify biomarkers related to cardiac dysfunction in the patients presenting myocardial injury (Table 4). Figure 2 displays these results jointly to ease their interpretation. Only the attributes that had a stability score larger than 900/1000 were selected. From this process, we obtained 17 attributes (20 if we take into consideration the significance at T1 and T2) for the myocardial injury study. For cardiac dysfunction, we obtained 22 from the patients who also presented myocardial injury.

### 3.3. Enrichment Analysis

All elements considered significant in the multiscale analysis were used as input for the enrichment analysis. The enrichment analysis with impala for transcripts and myocardial injury has yielded an overlap with the inactivation of the anaphase-promoting complex (APC/C) via direct inhibition of the APC/C complex pathway (*p*-value ≪ 0.05 with non-significant *q*-value). The same analysis for proteins and cardiac injury has shown a significant gene overlap with the complement and coagulation cascades. In particular, the coagulation factor XIII A chain (P00488), the complement C8 gamma chain (P07360), and the coagulation factor XIII B chain (F13B) are significant with *p*-value ≪ 0.005 and *q*-value 0.014. An enrichment analysis with Enrichr has shown that the genes considered significant in relation to myocardial injury are related to the pathways listed in Table 5.

The enrichment analysis for cardiac dysfunction in patients with myocardial injury with Impala has shown that catenin alpha 3 is related to arrhythmogenic right ventricular cardiomyopathy (*p*-value = 0.01 with non-significant *q*-value). The same analysis for the significant proteins yielded a significant overlap with the clotting cascade (*p*-value ≪ 0.05 and *q*-value = 0.04) and fibrin clot formation (*p*-value = 0.05 and *q*-value = 0.06) pathways. Finally, the enrichment analysis with MetaboAnalyst has found L-carnitine to be relevant in the beta oxidation of long chain fatty acids (*p*-value = 0.0996).

### 3.4. Prediction of SC Phenotypes

The sets of biomarkers obtained in our FS phase were used to predict the risk of myocardial injury assessed with circulating levels of troponin and the risk of cardiac dysfunction in patients with myocardial injury, assessed with the ejection fraction or an inotrope requirement. LOOCV was applied on the cohort of patients selected to study. In this manner, as many models as patients for each target variable per time point were created to obtain a reliable and unbiased estimate of model performance. In our study we used a logistic regression classifier as the baseline for comparison. CART models were executed with a maximum tree depth of 5 and 10 trees were explored at a time. With respect to the SVC, C = 1. The results of this classification are reported in Table 6 and Table 7. The McNemar’s test concluded that the sensitivity and specificity of the predictions were the same as the one of the original data (*p*-value ≪ 0.05 for all models).

## 4. Discussion

The present prospective clinical investigation demonstrates that the application of a ML pipeline to circulating transcriptomics, proteomics, and metabolomics data in patients with septic shock has huge potential to predict both the risk of myocardial injury (assessed with circulating levels of troponin) and the risk of cardiac dysfunction (assessed with echocardiography-derived left ventricle ejection fraction or an inotrope requirement). To our knowledge, the present results are the first data linking serial cardiac and hemodynamic measurements using a ML pipeline of OMICS data. The advantage of such a pipeline lies in the fact that it can untangle relevant relations between different markers and related them to a particular outcome (i.e., cardiac injury and cardiac dysfunction) through a data-driven approach. Thus, the application of ML techniques can improve the standard methods used in the classical clinical practice to assess these relations. Even though ML approaches require large amounts of data from big cohorts of patients to draw conclusions, this paper shows that it is also possible to obtain sensible results even with scarce data.

These results are of great interest as they throw light on the hypothesis that the root causes of cardiomyocyte injury and cardiac dysfunction in patients with septic shock may be approached using a ML pipeline tailored for OMICS analysis.

We offer an explanation to reinforce that changes in complement and coagulation systems were associated with myocardial injury in patients with septic shock. These observations are coherent with data showing that the coagulation system and microthrombosis are the main causes of ischemic heart affection and myocardial injury [24]. Microthrombosis has also been shown to be a main source of respiratory dysfunction and architectural lung injury in other inflammatory diseases as ARDS [25].

Ischemia-reperfusion injury has been shown to play a role in sepsis-associated organ dysfunction [26]. In this setting, platelets are critical mediators of thrombo-inflammation and have been shown to contribute to an exaggerated ischemia-reperfusion injury response [27,28,29]. However, the mechanisms underlying vulnerability to ischemia-reperfusion injury in septic shock patients is not well defined, nor the role of platelets in the process of SC [30].

Changes in the coagulation system have also been associated with myocardial dysfunctions in patients with ischemic myocardial injury. Indeed, there is now ample evidence supporting the concept of cardiac injury causing local inflammation and increased activation of pro-coagulant processes in patients with STEMI [31]. Moreover, biomarkers of coagulation and inflammation have been shown to provide pertinent and relevant distinction of patients suffering from coronary diseases and ischemic heart failure. In this regard, it is worth asking the question whether there is a plausible mechanistic basis that would allow myocardial capillary endothelial dysfunction to worsen right and left ventricular function in patients with septic shock [32]. The idea behind the present interrogation is to treat myocardial injury in septic shock patients by targeting pathways that link inflammation and thrombosis. For instance, several studies demonstrated a reduction in ischemic and clinical events with early high dose statins [33]. The present hypothesis is in line with some animal studies demonstrating that changes in systemic haemodynamics, coronary perfusion pressure, myocardial function, and increased tumour protein 53 expression with apoptosis related to bacterial exotoxin cause cardiac dysfunction. Indeed, in vivo changes were significantly inhibited by pretreatment with simvastatin, which provide novel evidence for the pleiotropic mechanisms by which septicaemia causes myocardial depression and hint at a potential role for simvastatin as an inhibitor of apoptosis in sepsis [34]. In our opinion, this finding highlights that subendocardial and myocardial ischemia are key damages induced by inflammation and sepsis causing diastolic and systolic heart dysfunctions [35,36]. The fact that recent data suggest that diastolic dysfunction is more frequent and associated with prognosis than systolic dysfunction in SC, corroborates our finding, as diastolic dysfunction is the first functional alteration during myocardial ischemia.

The underlying cause of SC could be, also, a disorder in communication between the intracellular contractile apparatus and extracellular matrix, resulting in attenuation of the myocardial contraction. In this regard, the fact that selected biomarkers could predict myocardial injury with good accuracy may contribute to underline the main causes of this transitory contraction interruption observed for the heart during sepsis.

We could also observe an association of alpha-1-antichymotrypsin and serum paraoxonase/arylesterase with myocardial injury. A recent proteomic study showed that circulating alpha-1-antichymotrypsin level was higher in patients with myocardial injury compared with stable angina or healthy controls [37]. Alpha-1-antichymotrypsin can inhibit the activity of neutrophil cathepsin G and mast cell chymase and with this mechanism may act as a mediator of inflammatory processes [38]. Paraoxonase is an antioxidant bioscavenger, responsible for hydrolysing lipid peroxides and decreased serum paraoxonase/arylesterase activity were related to poor prognosis (30-day mortality) in patients with sepsis [39]. These circulatory markers suggest a link of inflammation and oxidative stress with myocardial injury in septic shock.

In defiance of data scarcity, this study casts light on the relation between different biomarkers that play a role in patients with septic shock and strengthens some of the hypothesis posed by other aforementioned works. This is an exploratory methodology that may be further exploited over larger cohorts to elucidate the association between OMICS data and the biomarkers of interest.

In summary, this study showed that ML methods, applied to circulating OMICS data, can give an accurate estimation of myocardial injury and cardiac dysfunction in septic shock patients. This approach was also useful to investigate septic cardiomyopathy at molecular level and to identify a role of complement, coagulation, and inflammation pathways in the pathophysiology of myocardial injury. Our results, obtained in a small sized cohort of septic shock patients, show that the analysis of circulating OMICS data with a ML pipeline is a valuable tool to conduct research in critically ill patients.

### Limitations

Circulating troponin can be influenced by age and comorbidities (such as heart failure and renal function) besides the burden of acute illness caused by septic shock. Hence using a fixed threshold of troponin at ICU admission to classify patients as having myocardial injury may lead to an overestimation of cases. However, in the current analysis, we focused on identifying biomarkers acquired at ICU simultaneously to troponin measurements. In addition, there is no validated method to choose a different cut-off in patients with impaired renal clearance. Furthermore, elevated troponin has been shown to be associated with increased mortality independently of renal failure and elevated creatinine [40]. It is possible that use of inotropes impacts OMICS results. However, our study was not designed to answer this question and our small cohort does not allow exploring the impact of inotropes independent of cardiac function. The primary aim of this study was to provide new insight into the mechanisms of the phenotypes of myocardial injury and cardiac dysfunction. Hence, we used a large range of biological intermediates covering multiple levels of information (transcripts, proteins, and metabolites), which is a novelty in the field. These analyses require important resources and are not easily available in a clinical setting. However, exploratory studies as our own are often the basis for other mechanistic studies aimed at identifying biomarkers for prediction of the phenotypes or risk stratification. The main limitation in this paper is related to the cohort size. On the one hand, the high rate of non-eligibility and exclusion due to the OMICS techniques constraints and the discard of the cardiogenic shock patients, reduces the significance with respect to the original cohort. On the other hand, there is a small amount of data available to implement a ML pipeline to ascertain the role of circulating OMICS for assessing cardiac dysfunction and injury during septic shock. This lack of data has also limited the exploitation of the full potential of ML-based approaches so that we had to apply simple yet powerful methods that perform well under these circumstances. In our case, we used logistic regression as a baseline, CART, and SVC. Nevertheless, in the light of the results obtained it is worth exploring and improving the pipeline presented here in future research with larger patient cohorts.

## 5. Conclusions

The present findings indicate that the application of a ML pipeline to circulating OMICS data in patients with septic shock has the potential to predict the risk of myocardial injury (assessed with circulating levels of troponin) and the risk of cardiac dysfunction (assessed with echocardiography-derived left ventricle ejection fraction). This study is part of the multicentre prospective observational trial “ShockOmics” (ClinicalTrials.gov Identifier NCT02141607) [12].

## Figures and Tables

**Table 1:** Patient characteristics at ICU admission. Part I. Data are presented as median (interquartile range) or number (percentage). BMI—body mass index.

	All Patients	No Injury	Injury	*p*-Value
	27	7 (26%)	20 (74%)	*-*
Demographic parameters				
Age (years)	70 (21)	63 (48)	72 (20)	0.314
Sex (female)	8 (30%)	2 (29%)	6 (30%)	1.000
BMI (kg/m^2^)	26 (6)	27 (7)	26 (7)	0.646
Co-morbidities				
Heart failure	1 (4%)	0 (0%)	1 (5%)	1.000
Coronary artery disease	4 (15%)	1 (14%)	3 (15%)	1.000
Peripheral vascular disease	1 (4%)	1 (14%)	0 (0%)	0.259
Stroke	2 (7%)	0 (0%)	2 (10%)	1.000
Arterial hypertension	12 (44%)	3 (43%)	9 (45%)	1.000
Diabetes	8 (30%)	0 (0%)	8 (40%)	0.068
Chronic renal failure	1 (4%)	0 (0%)	1 (5%)	1.000
Chronic lung disease	6 (22%)	1 (14%)	5 (25%)	1.000
Patients on beta-blockers	5 (19%)	1 (14%)	4 (20%)	1.000

**Table 2:** Patient characteristics at ICU admission. Part II. Data are presented as median (interquartile range) or number (percentage). NonCV SOFA was calculated by subtracting the points of cardiovascular (CV) dysfunction to the total SOFA score. AKI—acute kidney injury according to “kidney disease: improving global outcomes” classification; APACHE II—acute physiology and chronic health evaluation II score; bpm—beats per minute; GCS—Glasgow coma scale; fluid balance—from the last 24 h; hscTnT—high sensitive cardiac troponin T; INR—international normalised ratio; PEEP—positive end-expiratory pressure; SAS—sedation-agitation scale; SOFA sequential organ failure assessment score; SvcO_2_—central venous oxygen saturation.

	All Patients	No Injury	Injury	*p*-Value
	27	7 (26%)	20 (74%)	-
Cardiovascular parameters				
hscTnT (ng/mL)	33 (61)	10.1 (5.9)	47.7 (80.8)	<0.000
EF (%)	50 (20)	60 (18)	45 (20)	0.019
Heart rate (bpm)	100 (33)	107 (39)	99 (27)	0.533
Cardiac output (L/min)	5.9 (2.9)	7.0 (1.6)	5.0 (3.0)	0.045
Mean arterial pressure (mmhg)	59 (7)	61 (5)	59 (10)	0.893
Systolic arterial pressure (mmhg)	88 (19)	85 (18)	88 (18)	0.725
Diastolic arterial pressure (mmhg)	47 (5)	47 (5)	45 (5)	0.431
Patients on vasopressors	26 (96%)	6 (86%)	20 (100%)	0.259
Norepinephrine dose (ug/kg/min)	0.33 (0.48)	0.26 (0.33)	0.32 (0.59)	0.533
Patients on inotropes	8 (30%)	1 (14%)	7 (35%)	0.633
Dobutamine dose (ug/kg/min)	5.0 (4.4)	-	4.9 (5.2)	-
Patients receiving steroids	0 (0%)	0 (0%)	0 (0%)	-
Fluid balance (L/kg)	0.02 (0.05)	0.02 (0.05)	0.03 (0.04)	0.911
Lactate (mmol/L)	4.3 (2.9)	3.4 (2.5)	4.8 (3.3)	0.263
SvcO_2_ (%)	76 (11)	68 (9)	78 (12)	0.012
APACHE II	24 (8)	20 (9)	24 (7)	0.063
SOFA	12 (4)	11 (4)	13 (4)	0.162
NonCV SOFA	8 (4)	7 (4)	9 (3)	0.179
Temperature (°C)	37.6 (1.2)	38.0 (1.3)	37.5 (1.6)	0.400
Glucose (mg/dL)	169 (70)	167 (50)	170 (91)	0.766
GCS	5 (7)	6 (7)	5 (8)	0.978
SAS	2 (1)	2 (1)	3 (2)	0.935
Mechanical ventilation	23 (85%)	6 (86%)	17 (85%)	1.000
PEEP (cmH_2_O)	8 (3)	10 (1.3)	8.0 (4.5)	0.155
PaO_2_/FiO_2_ (mmhg)	170 (103)	184 (55)	160 (116)	0.607
Creatinine (mg/dL)	1.4 (1.0)	1.0 (0.9)	1.5 (0.9)	0.041
Renal replacement therapy	1 (4%)	0 (0%)	1 (5%)	1.000
AKI	16 (59%)	2 (29%)	14 (70%)	0.084
Urinary output (L/d/kg)	0.02 (0.02)	0.02 (0)	0.02 (0.02)	0.750
Bilirubin (mg/dL)	1.3 (1.3)	1.3 (0.8)	1.2 (1.4)	0.725
INR	1.3 (0.3)	1.3 (0.3)	1.4 (0.3)	0.766
Total leucocyte count (103/mm^3^)	12.7 (12.7)	10.5 (11.2)	14.5 (13.8)	0.145
C-reactive protein (mg/L)	210 (230)	267 (261)	197 (226)	0.850
Fibrinogen (mg/L)	4.7 (1.3)	4.7 (1.0)	4.6 (2.2)	1.000
Platelets (×10^3^/mm^3^)	168 (82)	195 (17)	138 (90)	0.033

**Table 3:** One-way ANOVA for the biomarkers related to myocardial injury in the whole cohort (MI: n_T1_ = 20 and n_T2_ = 19—no MI: n_T1_ = 7 and n_T2_ = 8).

ID	Name	Biomarker	Time	*p*-Value	*q*-Value
MADL1	Mitotic arrest deficient 2 like 1	Transcript	T1	<0.005	0.41
WDR76	WD repeat domain 76	Transcript	T2	<0.005	0.44
SNORD104	Small nucleolar RNA, C/D box 104	Transcript	T2	<0.005	0.50
AL035420.3	Non-coding sequence	Transcript	T2	<0.005	0.35
AC092807.3	Non-coding sequence	Transcript	T2	<0.005	0.35
P00488	Coagulation factor XIII A chain	Protein	T1	<0.005	0.06
P01011	Alpha-1-antichymotrypsin	Protein	T1	<0.005	<0.003
P05160	Coagulation factor XIII B chain	Protein	T1	<0.005	0.06
P27169	Serum paraoxonase/arylesterase 1	Protein	T1	<0.005	0.02
Q12805	EGF-containing fibulin-like extracellular matrix protein 1	Protein	T1	<0.005	0.06
P00488	Coagulation factor XIII A chain	Protein	T2	0.006	0.41
P01011	Alpha-1-antichymotrypsin	Protein	T2	<0.005	0.21
P06727	Apolipoprotein A-IV	Protein	T2	0.02	0.47
P07360	Complement component C8 gamma chain	Protein	T2	0.01	0.46
P13591	Neural cell adhesion molecule 1	Protein	T2	0.05	0.51
P27169	Serum paraoxonase/arylesterase 1	Protein	T2	<0.005	0.40
Q12805	EGF-containing fibulin-like extracellular matrix protein 1	Protein	T2	<0.005	0.41
PC ae C36:5	Phosphatidylcholine (1x O-acyl, 1x O-alkyl)	Metabolite	T1	0.009	0.12
PC ae C38:6	Phosphatidylcholine (1x O-acyl, 1x O-alkyl)	Metabolite	T1	<0.005	0.12
Total DMA	Dimethylarginine	Metabolite	T2	0.01	0.24

**Table 4:** One-way ANOVA for the biomarkers related to cardiac dysfunction in patients presenting myocardial injury (MI with dysfunction: n_T1_ = 13 and n_T2_ = 9—MI without dysfunction: n_T1_ = 7 and n_T2_ = 10).

ID	Name	Biomarker	Time	*p*-Value	*q*-Value
ZNF474	Zinc finger protein 474	Transcript	T1	<0.005	0.19
ANP32BP1	Acidic nuclear phosphoprotein 32 family member B pseudogene 1	Transcript	T1	<0.005	0.19
SERBP1P6	SERPINE1 mRNA binding protein 1 pseudogene 6	Transcript	T1	<0.005	0.44
TMPRSS12	Transmembrane serine protease 12	Transcript	T1	<0.005	0.44
LINC02489	Long intergenic non-protein coding RNA 2489	Transcript	T1	<0.005	0.44
SDS	Serine dehydratase	Transcript	T1	<0.005	0.44
KRT89P	Keratin 89 pseudogene	Transcript	T1	<0.005	0.44
AC073534.2	Non-coding sequence	Transcript	T1	<0.005	0.44
HOTAIRM1	HOXA transcript antisense RNA, myeloid-specific 1	Transcript	T1	<0.005	0.44
AL627309.2	Non-coding sequence	Transcript	T1	<0.005	0.44
CTNNA3	Catenin alpha 3	Transcript	T2	<0.005	0.01
A2ML1	Alpha-2-macroglobulin like 1	Transcript	T2	<0.005	0.02
P00748	Coagulation factor XII	Protein	T1	0.02	0.99
P07359	Platelet glycoprotein Ib alpha chain	Protein	T1	0.03	0.99
P27169	Serum paraoxonase/arylesterase 1	Protein	T2	0.04	0.99
Q9ULI3	Protein HEG homolog 1	Protein	T2	0.04	0.99
Cit	Citrulline	Metabolite	T1	0.05	0.93
PC aa C40:3	Phosphatidylcholine (2x O-acyl)	Metabolite	T1	0.05	0.93
Val	Valine	Metabolite	T2	0.03	0.93
Met	Methionine	Metabolite	T2	0.04	0.93
C0	Carnitine	Metabolite	T2	0.05	0.93
t4 OH Pro	trans-4-Hydroxyproline	Metabolite	T2	0.05	0.93

**Table 5:** Enrichment analysis ranking pathways based on significant overlaps between given genes and annotated gene sets.

Pathway	Adjusted *p*-Value
Mitotic Spindle Checkpoint Homo sapiens R-HSA-69618	0.0374
APC/C pathway	0.0374

**Table 6:** Classification results for myocardial injury in the whole cohort.

Data	Method	Accuracy	Sensitivity	Specificity
T1	Logistic	0.33	0.35 (0.15, 0.59)	0.29 (0.04, 0.71)
T2	Logistic	0.81	0.83 (0.59, 0.96)	0.75 (0.35, 0.97)
T1	CART	0.44	0.55 (0.32, 0.77)	0.14 (0.00, 0.58)
T2	CART	0.85	0.89 (0.65, 0.99)	0.75 (0.35, 0.97)
T1	SVC	1.00	1.00 (0.83, 1.00)	1.00 (0.29, 1.00)
T2	SVC	1.00	1.00 (0.81, 1.00)	1.00 (0.63, 1.00)

**Table 7:** Classification results for cardiac dysfunction in patients with myocardial injury.

Data	Method	Accuracy	Sensitivity	Specificity
T1	Logistic	0.63	0.58 (0.28, 0.85)	0.67 (0.38, 0.88)
T2	Logistic	0.68	0.67 (0.35, 0.90)	0.70 (0.35, 0.93)
T1	CART	0.63	0.50 (0.21, 0.79)	0.73 (0.45, 0.92)
T2	CART	0.68	0.58 (0.28, 0.85)	0.80 (0.44, 0.97)
T1	SVC	1.00	1.00 (0.54, 1.00)	1.00 (0.75, 1.00)
T2	SVC	1.00	1.00 (0.63, 1.00)	1.00 (0.63, 1.00)

## Data Availability

As per Swiss law, data from Swiss patients cannot be shared publicly unless the patient gave his/her consent. Data can potentially be available upon request pending request and authorization from the Ethics Committee. Requests for data should be sent to the corresponding author, who will contact the Geneva Cantonal Ethics Committee with the request (ccer@etat.ge.ch, Rue Adrien-Lachenal 8, 1207 Geneva, Switzerland).

## Supplementary Materials

The following are available online at https://www.mdpi.com/article/10.3390/jcm10194354/s1. Figure S1. Patient flow. HE—Hôpital Erasme (Brussels, Belgium); HUG—Geneva University Hospitals (Geneva, Switzerland); N—Number of patients. Table S1. STROBE Statement Checklist. List of items that should be included in reports of observational studies according to the STROBE Recommendations.
