# Supplementary figures and images for: Application of an Exploratory Knowledge-Discovery Pipeline Based on Machine Learning to Multi-Scale OMICS Data to Characterise Myocardial Injury in a Cohort of Patients with Septic Shock: An Observational Study
Source: J Clin Med. 2021 Sep 24;10(19):4354. doi: 10.3390/jcm10194354 (PMC8509561; doi:10.3390/jcm10194354)

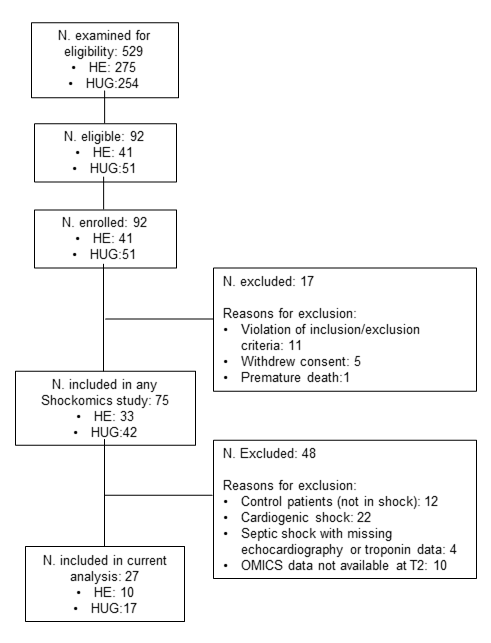

Supplement: Supplementary file 1 [file jcm-10-04354-s001.zip › S1 Fig.tif]
